# Investigation of Dried Blood Spot Cards for Fatty Acid Analysis Using Porcine Blood

**DOI:** 10.1155/2021/6624751

**Published:** 2021-08-28

**Authors:** Jordan Wood, Larry J. Minter, Michael K. Stoskopf, Doug Bibus, Dempsy Ange, Troy N. Tollefson, Vivek Fellner, Kimberly Ange-van Heugten

**Affiliations:** ^1^Department of Animal Science, North Carolina State University, Raleigh, NC 27695, USA; ^2^North Carolina Zoo, 4401 Zoo Pkwy, Asheboro, NC 27205, USA; ^3^Department of Clinical Sciences, College of Veterinary Medicine, North Carolina State University, 1060 William Moore Dr, Raleigh, NC 27607, USA; ^4^Lipid Technologies LLC, P.O. Box 216, Austin, MN 55912, USA; ^5^Beech Ridge Pork Farm Inc., Belhaven, NC 27810, USA; ^6^Mazuri® Exotic Animal Nutrition, PMI Nutrition, Land O' Lakes, Inc.,, St. Louis, MO 63166, USA

## Abstract

Fatty acids, especially omega-3 and omega-6 fatty acids, are important for reproductive and cardiovascular health in animals. While monitoring fatty acids is traditionally conducted using frozen blood fractions such as serum and plasma, advancements in analytical technology have developed a method of collecting microsamples of dried whole blood on Ahlstrom 226 grade filter paper that can provide information on long-term fatty acid status of animals. Blood samples were collected from five male pigs in both the traditional frozen method and on dried blood spot cards (DBS). The DBS samples were collected with untreated syringes and tubes, and approximately 320 *μ*L of blood was placed on each card with approximately 80 *μ*L per spot (4 spots). Statistical analysis was performed to compare the two sample groups to each other using the Mann–Whitney *U*-test and determine if DBS samples were similar to traditional whole blood samples. Of the 30 fatty acids and fatty acid groups with measurable concentrations, only four individual fatty acids, behenic acid, omega-3 docosapentaenoic acid, nervonic acid, and adrenic acid, had statistical differences. Most of these differences were minor and could be due to analytical errors or contamination. Comparisons between sample types found similar concentrations of key omega fatty acids and PUFAs and support the use of DBS collection as a less invasive method of blood collection and fatty acid analysis.

## 1. Introduction

Fatty acids are critical dietary components that support the general health of animals. The omega-3 and omega-6 fatty acids in particular help to maintain cellular membrane structure and function, support the immune response, and can impact the reproductive and cardiovascular health of an animal [1–3]. In humans, supplementation of dietary polyunsaturated fatty acids such as eicosapentaenoic acid (EPA) and docosahexaenoic acid (DHA) has been used to treat disorders such as elevated triglycerides, insulin resistance, and metabolic inflammation [4]. Additionally, an inverse relationship exists between dietary and circulating EPA and DHA and the prevalence of cardiovascular disease, with higher levels of EPA and DHA reducing the risk of cardiovascular disease [5]. Blood measures of various biomarkers, including fatty acids, are useful in determining optimal dietary intake of nutrients in animals [6, 7]. Often, these blood biomarkers are analyzed in serum or plasma; however, whole blood has been found to reflect long-term fatty acid status compared to the short-term status (days to months) provided by serum or plasma concentrations [8, 9].

Advancements in analytical methods have improved over the years, and the ability to analyze fatty acids and other biomarkers with microsamples has been of interest for a variety of species [10]. One type of the fatty acid microsample method uses specially prepared filter paper and small drops of whole blood collected through traditional blood draws. Whole blood drops are allowed to dry on the cards and can then be used for analyzing both disease and nutrient markers, including fatty acids [10, 11]. The use of dried blood spot (DBS) cards for whole blood collection is of particular interest in situations where the proper storage of whole blood is not possible such as field work. While DBS methods have been validated for many assays in humans, there are few resources available to validate DBS in other species [12].

The goal of this research was to evaluate DBS as suitable for measurement of animal whole blood fatty acids. It also provides limited data on whole blood DBS samples for weanling swine. We compared the results from whole blood and DBS samples sent within one week of collection for analysis.

## 2. Materials and Methods

Whole blood was collected in fall 2017 from five unrelated castrated domestic pigs (*Sus scrofa domesticus*) weighing between 6.8 and 9.1 kg at weaning. Prior to weaning, the pigs were maintained on a diet of their sow's milk and the sows were fed a corn and soybean meal base mix lactation diet. Blood was obtained from each pig's jugular vein during regular veterinary wellness examinations during weaning. Blood was collected in untreated syringes and tubes and directly transferred to PerkinElmer Spot Saver Cards (PerkinElmer Waltham, MA) after collection. These cards are made of Ahlstrom 226 grade filter paper which has been tested and verified to meet the specifications for both the Center of Disease Control (CDC) and the Food and Drug Administration (FDA) for dried blood collection. These specifications include the filter paper being 100% pure cotton fiber with no wet-strength additives, a pH between 5.7 and 7.5 and no more than 0.1% ash content [13]. These specifications ensure consistency across labs and tests and lend to their biostability. Each of the four spots on a blood card received approximately 80 *μ*L of blood for a total of 320 *μ*L per animal per card for 4 of the pigs. Blood from the 5^th^ pig was applied to all the spots on 3 cards. A single blood spot is sufficient for fatty acid analyses, but extra spots collected in case spots were of poor quality or contaminated. These cards were kept at room temperature until shipment to NC State University. A control, heparinized whole blood sample was also collected from each animal for analysis and frozen at −80°C. All five frozen heparinized samples and five spot cards were sent to Lipid Technologies (Austin, MN) for a full fatty acid profile within a week of collection. The additional two cards from pig 5 were kept at room temperature (approximately 23°C) for one year and two years, respectively, before being sent to Lipid Technologies for analysis.

A comparison between the control whole blood (WB) samples (*n* = 5) and the dried blood spots (DBS) (*n* = 5) was conducted through manual calculations of nonparametric statistics using the Mann–Whitney *U*-test with *α* = 0.05 to determine if WB differed from DBS. Additionally, differences were visually assessed between the WB, initial DBS, and both the one- and the two-year held DBS samples from pig 5.

## 3. Results

Of the 36 individual fatty acids and 10 fatty acid groups analyzed, 20 individual fatty acids and all 10 groups had measurable concentrations (Table 1). When comparing WB to DBS across all five individual pigs, four fatty acids had statistically significant differences. Whole blood was lower than DBS for behenic acid (22 : 0), docosapentaenoic (Osbond) acid (DPA, 22 : 5w6), and nervonic acid (24 : 1) but was higher than DBS for adrenic acid (22 : 4w6).

Visual differences were seen for most fatty acids and acid groups when comparing the one-and two-year stored samples from pig 5 to the initial DBS and whole blood samples from this same animal. These differences are based solely on qualitative comparisons between the concentrations seen in all sample groups. No statistical analysis was appropriate as samples were only available from a single animal.

## 4. Discussion

The four individual fatty acids that had statistical differences between DBS and WB samples were all low concentration lipids and except for the behenic acid were driven by fairly large proportional differences in only one or two samples, suggesting minor laboratory handling errors or issues when placing the samples on the cards during collection The consistently higher values for card origin samples for behenic acid may suggest interference from the DBS card fibers or an environmental contaminant. Unfortunately, no blank cards were run as negative controls as previous testing by the developers and standards set by the CDC and FDA did not indicate the need [13]. If contamination did occur, it is likely that it happened during collection in the animal enclosure as opposed to having contaminated cards. However, control cards will be considered for future research. Overall, our study provides strong evidence for the use of DBS collection as an alternative to liquid WB collection for swine when samples can be analyzed soon after draw. DBS can be used to efficiently sample in difficult free-ranging field conditions and for animals that cannot provide large blood volume samples for any reason. These DBS samples can also be easier to ship as they can remain at ambient temperatures, the drying process leads to reduced pathogen loads, and shipments of dried samples are considered exempt nonregulated materials in many regions [10, 14]. These factors can reduce the total cost of sample collection and management.

The few differences in the fatty acid compositions of these sample types is encouraging for fatty acid research, especially those involving omega fatty acids and PUFAs as there were no differences for the key fatty acids in these categories. Fatty acids that are also the most subject to degradation [15], EPA, DHA, and arachidonic acid, did not have statistically significant differences. This means that DBS samples should provide a faster collection alternative with comparable results to traditional whole blood collections.

When looking at storage time as a factor in the DBS samples from pig 5, there were potential differences noted between the original “fresh” sample and the ones stored for 1 or 2 years at room temperature. Due to the low *n*, this could not be statistically analyzed. The collection of these extended time samples was not the goal of this project; however, further research into the DBS card validity via time postsampling and storage techniques may be needed.

While this dataset is small and preliminary in breadth, it provides valuable information for the fields of comparative nutrition studies. The use of DBS cards for whole blood fatty acids is currently being investigated in exotic species where large blood samples, large sample populations, and blood sample processing (or temperature management) may not be available. Currently, there are studies that have used DBS with positive results to analyze managed and free-ranging eastern box (*Terrapene carolina carolina*) and common snapping (*Chelydra serpentina*) turtles [16] and in free-ranging green turtles (*Chelonia mydas*) and Kemp's ridley turtles (*Lepidochelys kempii*) [17]. By utilizing swine as a domestic model for exotic mammals, this dataset has provided strong evidence that DBS can be utilized for exotic mammalian samples and reptile samples.

## 5. Conclusion

Comparisons between liquid whole blood and DBS samples support the use of DBS collection as a less invasive method of blood collection and fatty acid analysis. Fatty acid concentrations analyzed were similar between the two collection methods including key omega fatty acids and PUFAs that are critical in cardiovascular and general health in many species. The four fatty acids with differences should be analyzed with caution, and the question of behenic acid should be further investigated. Differences in the stored DBS samples collected from pig 5 suggest a finite storage time and warrant further study to evaluate temperature and humidity storage conditions on sample stability.

## Figures and Tables

**Table 1:** Fatty acid (% of total fat) profile of liquid whole blood (WB) (*n* = 5) and dried blood spots (DBS) (*n* = 5) of 5 pigs with a Mann–Whitney *U*-test of WB to DBS samples^1^.

Type	DBS								WB					
Pig ID	1	2	3	4	5	Average	5^2^	5^3^	1	2	3	4	5	Average
*Fatty acid*													
Myristic acid (14 : 0)	1.00	1.25	1.35	1.33	0.98	**1.18**	**0.87**	**0.66**	1.25	1.04	1.40	1.39	1.05	**1.23**
Palmitic acid (16 : 0)	25.75	25.68	25.01	26.24	22.34	**25.00**	**24.14**	**25.14**	25.68	26.10	25.64	27.70	23.49	**25.72**
Palmitoleic acid (16 : 1w7)	4.44	4.19	4.25	5.10	6.40	**4.88**	**5.92**	**5.95**	4.40	3.99	4.29	4.64	6.57	**4.78**
Stearic acid (18 : 0)	7.65	9.05	8.63	8.43	7.28	**8.21**	**10.56**	**9.55**	7.94	9.37	8.47	8.66	6.89	**8.27**
Oleic acid (18 : 1w9)	22.41	20.50	24.56	24.92	22.06	**22.89**	**24.45**	**26.56**	22.98	20.30	24.44	24.11	21.85	**22.74**
Linoleic acid (18 : 2w6)	27.65	28.49	24.51	23.70	29.47	**26.76**	**25.20**	**23.82**	27.08	28.66	24.30	23.89	29.53	**26.69**
*γ*-Linolenic acid (18 : 3w6)	0.29	0.28	0.38	0.27	0.30	**0.30**	**0.30**	**0.00**	0.23	0.26	0.34	0.20	0.33	**0.27**
Arachidic acid (20 : 0)	0.16	0.19	0.15	0.08	0.00	**0.12**	**0.11**	**0.00**	0.21	0.15	0.12	0.07	0.00	**0.11**
Paullinic acid (20 : 1w7)	0.11	0.24	0.24	0.12	0.17	**0.18**	**0.15**	**2.34**	0.11	0.21	0.25	0.09	0.21	**0.17**
*α*-Linolenic acid (18 : 3w3)	0.53	0.58	0.56	0.51	0.58	**0.55**	**0.50**	**0.40**	0.56	0.51	0.52	0.42	0.59	**0.52**
Eicosenoic acid (20 : 2w6)	0.27	0.27	0.25	0.15	0.32	**0.25**	**0.29**	**0.29**	0.28	0.26	0.29	0.21	0.23	**0.25**
h-*γ*-Linolenic acid (20 : 3w6)	0.35	0.45	0.33	0.38	0.41	**0.38**	**0.32**	**0.28**	0.33	0.40	0.30	0.32	0.36	**0.34**
Arachidonic acid (20 : 4w6)	6.19	6.12	6.78	5.85	6.90	**6.37**	**4.16**	**3.39**	6.34	6.17	6.93	5.86	6.38	**6.34**
EPA (20 : 5w3)	0.18	0.20	0.17	0.18	0.16	**0.18**	**0.15**	**0.22**	0.18	0.20	0.15	0.14	0.17	**0.17**
Behenic acid (22 : 0)	0.31	0.29	0.25	0.41	0.30	**0.31** ^**a**^	**0.48**	**0.32**	0.03	0.07	0.04	0.08	0.08	**0.06** ^**b**^
Adrenic acid (22 : 4w6)	0.49	0.47	0.50	0.52	0.48	**0.49** ^**a**^	**0.26**	**0.06**	0.52	0.54	0.61	0.55	0.51	**0.55** ^**b**^
DPA (Osbond acid) (22 : 5w6)	0.32	0.34	0.32	0.39	0.36	**0.35** ^**a**^	**0.13**	**0.08**	0.24	0.32	0.26	0.34	0.30	**0.29** ^**b**^
DPA (clupanodonic acid) (22 : 5w3)	0.69	0.59	0.60	0.58	0.51	**0.59**	**0.38**	**0.25**	0.56	0.65	0.56	0.61	0.49	**0.57**
DHA (22 : 6w3)	1.02	0.66	1.02	0.62	0.86	**0.84**	**0.55**	**0.19**	0.98	0.71	0.96	0.62	0.92	**0.84**
Nervonic acid (24 : 1)	0.19	0.18	0.14	0.20	0.10	**0.16** ^**a**^	**0.15**	**0.10**	0.11	0.09	0.12	0.10	0.05	**0.09** ^**b**^
Saturates	34.87	36.46	35.40	36.50	30.91	**34.83**	**36.15**	**35.66**	35.11	36.73	35.68	37.89	31.52	**35.39**
Monoenes	22.70	20.91	24.94	25.25	22.33	**23.23**	**25.38**	**29.04**	23.20	20.60	24.81	24.30	22.11	**23.00**
PUFA	37.98	38.44	35.41	33.15	40.36	**37.07**	**32.55**	**29.35**	37.29	38.68	35.22	33.17	39.80	**36.83**
HUFA	9.24	8.83	9.71	8.53	9.68	**9.20**	**6.26**	**4.69**	9.14	9.00	9.78	8.44	9.12	**9.10**
Total w3	2.43	2.03	2.35	1.88	2.11	**2.16**	**1.73**	**1.42**	2.28	2.08	2.19	1.79	2.16	**2.10**
Total w6	35.55	36.41	33.07	31.27	38.25	**34.91**	**30.66**	**27.93**	35.01	36.60	33.04	31.37	37.64	**34.73**
Total w9	22.59	20.68	24.70	25.12	22.16	**23.05**	**25.23**	**26.70**	23.09	20.39	24.56	24.21	21.90	**22.83**
w6/w3	14.65	17.93	14.10	16.60	18.12	**16.28**	**17.75**	**19.62**	15.34	17.60	15.12	17.50	17.41	**16.59**
Omega-3 HUFA	20.48	16.44	18.37	16.17	15.80	**17.45**	**19.62**	**18.61**	18.83	17.45	17.05	16.25	17.22	**17.36**
Omega-6 HUFA	79.52	83.56	81.63	83.83	84.20	**82.55**	**80.38**	**81.39**	81.17	82.55	82.95	83.75	82.78	**82.64**

^1^Differing superscripts (^a, b^) in averages columns are significantly different at (*p* < 0.05) for DBS compared to WB. ^2^Sample represents the 1-year held DBS card at room temperature and is not included in the DBS average. ^3^Sample represents the 2-year held DBS card at room temperature and is not included in the DBS average.

## Data Availability

The data used to support the findings of this study are included within the article.

## References

[1] Fritsche K. (2006). Fatty acids as modulators of the immune response. *Annual Review of Nutrition*.

[2] Mozaffarian D., Wu J. H. Y. (2011). Omega-3 fatty acids and cardiovascular disease: effects on risk factors, molecular pathways, and clinical events. *Journal of the American College of Cardiology*.

[3] Saker K. E., Eddy A. L., Thatcher C. D., Kalnitsky J. (1998). Manipulation of dietary (*N* − 6) and (*N* − 3) fatty acids alters platelet function in cats. *Journal of Nutrition*.

[4] Silva Figueiredo P., Carla Inada A., Marcelino G. (2017). Fatty acids consumption: the role metabolic aspects involved in obesity and its associated disorders. *Nutrients*.

[5] Connor W. E. (2000). Importance of *n* − 3 fatty acids in health and disease. *American Journal of Clinical Nutrition*.

[6] Clauss M., Dierenfeld E. S., Bigley K. E. (2008). Fatty acid status in captive and free-ranging black rhinoceroses (*Diceros bicornis*). *Journal of Animal Physiology and Animal Nutrition*.

[7] Wood J., Koutsos E., Kendall C. J. (2020). Circulating nutrients and hematological parameters in managed African elephants (*Loxodonta Africana*) over a 1‐year period. *Zoo Biology*.

[8] Baylin A., Kim M. K., Donovan-Palmer A. (2005). Fasting whole blood as a biomarker of essential fatty acid intake in epidemiologic studies: comparison with adipose tissue and plasma. *American Journal of Epidemiology*.

[9] Risé P., Eligini S., Ghezzi S., Colli S., Galli C. (2007). Fatty acid composition of plasma, blood cells and whole blood: relevance for the assessment of the fatty acid status in humans. *Prostaglandins, Leukotrienes and Essential Fatty Acids*.

[10] Freeman J. D., Rosman L. M., Ratcliff J. D., Strickland P. T., Graham D. R., Silbergeld E. K. (2018). State of the science in dried blood spots. *Clinical Chemistry*.

[11] Armstrong J. M., Metherel A. H., Stark K. D. (2008). Direct microwave transesterification of fingertip prick blood samples for fatty acid determinations. *Lipids*.

[12] Bailey-Hall E., Nelson E. B., Ryan A. S. (2008). Validation of a rapid measure of blood PUFA levels in humans. *Lipids*.

[13] Davin B., Hannon W. H. (2014). Dried blood spot cards. *Dried Blood Spots: Applications and Techniques*.

[14] Mei J. (2014). Dried blood spot sample collection, storage, and transportation. *Dried Blood Spots: Applications and Techniques*.

[15] Ismail A., Bannenberg G., Rice H. B., Schutt E., MacKay D. (2016). Oxidation in EPA‐and DHA‐rich oils: an overview. *Lipid Technology*.

[16] Dass K., Koutsos E., Minter L. J., Ange-van Heugten K. (2020). Analysis of fatty acid profiles in eastern box (*Terrapene Carolina Carolina)* and common snapping *(Chelydra serpentine)* turtles for wild and in-human care environments. *Journal of Zoo and Wildlife Medicine*.

[17] Koutsos E., Minter L. J., Ange-van Heugten K. D., Mejia-Fava J., Harmes C. (2021). Blood fatty acid profiles of juvenile wild green turtles (Chelonia mydas) and kemp’s ridley turtles (*Lepidochelys kempii*). *Journal of Zoo and Wildlife Medicine*.

